# Revolutionizing Allergy Care: Sublingual Immunotherapy for House Dust Mite Allergy

**DOI:** 10.7759/cureus.95013

**Published:** 2025-10-20

**Authors:** Taha A Qureshi, Sitesh Roy, Gautam Modi, Neeraj Gupta, Gayatri Pandit, Devesh K Joshi, Monil Gala

**Affiliations:** 1 Allergy, Jawahar Lal Nehru Memorial Hospital, Srinagar, IND; 2 Allergy, Dr. Roy Health Solutions Clinic, Mumbai, IND; 3 Allergy, Modi Allergy Clinic, Patna, IND; 4 Pediatrics and Allergy, Sir Ganga Ram Hospital, New Delhi, IND; 5 Allergy, Samarth ENT and Allergy Centre, Bangalore, IND; 6 Medical Affairs, Dr. Reddy's Laboratories Ltd., Hyderabad, IND

**Keywords:** allergic rhinitis, asthma, dermatophagoides, dust mites, sublingual immunotherapy

## Abstract

House dust mite (HDM) allergies affect a significant portion of the global population. A considerable number of people in India suffer from allergic rhinitis (AR), and many individuals with AR are affected by HDM allergies. The management of allergic conditions, such as AR and allergic asthma (AA), has predominantly depended on drugs providing symptomatic relief and avoidance measures. These approaches do not include disease-modifying drugs and have limitations, including incomplete relief, slow onset of action, lack or drop-off in efficacy with continued use, and frequent exacerbations. With multiple species of HDM in India and high rates of sensitization among patients with nasobronchial allergy, symptomatic management is inadequate. Allergen immunotherapy (AIT), including sublingual immunotherapy (SLIT), represents the only disease-modifying treatment for allergic conditions. Unlike subcutaneous immunotherapy (SCIT), SLIT has the advantages of being non-invasive, allowing at-home administration, and having a lower potential for anaphylactic reactions. This review highlights the need for SLIT and explores the mechanism and clinical benefits of this novel immunotherapy.

## Introduction and background

Sublingual immunotherapy (SLIT) for the management of allergic conditions involves introducing allergens under the tongue, with the aim of inducing immune tolerance. It is a substitute for subcutaneous immunotherapy (SCIT), often referred to as "allergy shots." SLIT is commonly used to treat allergic rhinitis (AR) and allergic asthma (AA) by slowly desensitizing the immune system to specific allergens. The therapy is given in oral tablet or drop form, with daily administration of allergen extracts, which is convenient and less invasive than injections. SLIT has been shown to improve patients' quality of life (QoL), reduce symptoms, and decrease the need for medication. It is considered safe overall, with mild side effects such as oral itching, swelling, or throat irritation being the most common. However, it requires strict adherence to dosing schedules for long-term efficacy [1,2].

House dust is a known trigger of asthma and allergic rhinoconjunctivitis, and specifically, house dust mites (HDMs) are the main allergenic source in house dust. HDMs are an established cause of allergies, leading to AA, AR, conjunctivitis, atopic dermatitis (AD), and, in rare cases, anaphylaxis through the consumption of dust mite-contaminated food [3,4].

HDMs can be found almost worldwide, and it is estimated that HDM allergy affects 1%-2% of the global population - approximately 65-130 million people. HDMs are microscopic arthropods found in indoor environments. They thrive in warm, humid conditions and are significant contributors to indoor allergens. Their primary allergenic components are proteins found in their fecal matter, body parts, and secretions, which can trigger allergic diseases such as AR, AA, and AD in sensitized individuals. Globally, *Dermatophagoides pteronyssinus* and *Dermatophagoides farinae* are the most important allergen sources for patients with HDM allergies. Storage mites, including *Acarus siro*, *Blomia tropicalis*, *Glycyphagus domesticus*, *Lepidoglyphus destructor*, and *Tyrophagus putrescentiae*, are also found in indoor environments, especially in warm, humid climates [5-7].

HDM allergy is a type I hypersensitivity reaction mediated by immunoglobulin E (IgE) in response to allergens present in mite bodies and/or fecal pellets. Inhalation of the proteins found in mite fecal pellets and/or mite bodies leads to contact with the airway epithelium, wherein epithelial cells are bonded together by adherens and tight junctions. Patients who are allergic to HDM have IgE antibodies that show broad cross-reactivity between homologous Der p and Der f (*D. pteronyssinus* and *D. farinae* allergens). This cross-reactivity is attributed to high sequence and structural similarities between these allergens [8,9]. It is important to note that the Der p 1 allergen can disrupt tight junctions in the modified bronchial epithelial barrier, thus modifying its permeability. This increases the access of allergens to dendritic cells (DCs) and can lead to the development of asthma [10].

*D. pteronyssinus* is one of the most common species of HDMs and is a major source of allergens in indoor environments. Its allergenic proteins, mainly Der p 1 and Der p 2, play an important role in inducing allergic diseases like AA, AR, and AD. These allergens are proteolytic enzymes that can break the epithelial barrier and induce immune responses [9]. Exposure to *D. pteronyssinus* allergens results in sensitization, whereby a person develops IgE antibodies against the proteins produced by the mite. This results in chronic inflammatory conditions through the mediation of T helper 2 (Th2) cells, eosinophils, and other immune cells. *D. pteronyssinus* allergens are predominantly prevalent in areas with warm and humid climates because of the proliferation of mites. *D. farinae* is also a major source of indoor allergens worldwide. Microscopic HDMs feed on human skin scales as well as organic debris and thrive in humid environments. Allergens from *D. farinae* and *D. pteronyssinus* are found in their feces, exoskeletons, and secretions [6,9,10].

This article aims to highlight the burden of HDM allergy in India and reviews the evidence on SLIT for the management of HDM allergy. In addition, the current status of SLIT in India, with respect to guidelines and regulatory status, is discussed.

## Review

Prevalence of HDM in India

Dust mites have been recognized as an important sensitizer in the Indian population, with numerous studies elaborating on the prevalence of different allergenic dust mites. India tops the list of the highest recorded HDM diversity, with 12 species [7,11]. A study by Mondal et al. reported the prevalence of HDM in an urban location in eastern India. Dust samples collected from the homes of 43 patients with AR, bronchial asthma, AD, and conjunctivitis were positive for the presence of acarine fauna, including *D. pteronyssinus*, *D. farinae*, *B. tropicalis*, *A. siro*, *L. destructor*, and *T. putrescentiae*. Dust from the homes of patients had a significantly higher average number of all mites per gram of dust compared with homes of controls (275.74 ± 37.70 vs. 44.66 ± 12.89, respectively) [12]. Other studies in urban India revealed the most prevalent HDM to be *D. pteronyssinus*, with frequencies of 72.5%, 61.74%, and 100%, respectively, from studies conducted in Vadodara (Gujarat), Patiala (Punjab), and Kolkata (West Bengal) [13-15].

Sensitization to HDM is also reported to be high in various studies conducted in India. One study reported that 96% of patients with nasobronchial allergy showed sensitization to HDM [16]. Mondal et al. reported that a significant positive skin prick test (SPT) response was noted in 88.7% of patients (toward either house dust or HDM allergens), and sensitization to *D. pteronyssinus* was 81.21%, to *D. farinae* was 87.87%, and to *B. tropicalis* was 74.24% [12]. Interestingly, it has been reported that “blockers” have significantly higher rates of sensitization to HDM than “sneezers and runners” (40% vs. 15%, p = 0.05; 28.7% vs. 16.3%, p = 0.004, from a study in Kolkata) [17,18]. Sensitization to storage mites is lower than that of HDM. Studies in India report sensitization to *A. siro* to be 9%-48.8%, that of *L. destructor* is 25.15%-42.1%, and that of *T. putrescentiae* is 18.78%-48.1%. These rates are lower than the rate of sensitization to *D. pteronyssinus* (40%-81.21%) and *D. farinae* (40%-87.87%) [7]. However, for *B. tropicalis*, also classified as a storage mite, sensitization rates between 32% and 72% have been identified across various regions of India [11,19].

Taken together, exposure to these allergens is a major risk factor for AR, AA, and AD. These immunological reactions are mediated by IgE pathways, leading to symptoms of allergy, including nasal congestion, wheezing, and skin irritation [17].

Management of allergic conditions

Allergen avoidance is considered an important part of therapy to achieve a reduction in exposure through altering conditions that would otherwise permit growth of HDM [7]. These measures include increasing ventilation, decreasing humidity, and frequent washing of bed linen in hot water [17]. However, allergens in locations other than homes can also cause allergies. Therefore, several pharmacotherapy options have been developed for the management of allergic conditions. Modalities for symptomatic therapy include oral or intranasal antihistamines and corticosteroids, oral leukotriene antagonists, and inhaled β2-agonists and corticosteroids. However, these modalities are plagued with limitations, including sometimes incomplete relief, slow onset of action, lack or drop-off in efficacy with continued use, suboptimal adherence, and frequent exacerbations [20,21]. Allergy immunotherapy (AIT) is a treatment designed to reduce the symptoms of allergic reactions by altering the body's immune response. Regular administration of increasing doses of an allergen can desensitize the immune system to the allergen, thus preventing or reducing allergic symptoms with long-term treatment [22]. SLIT is a form of allergy treatment where allergen extracts are administered under the tongue to promote desensitization to specific allergens. SLIT offers a non-invasive alternative to SCIT [1,23]. AIT is the only available modality of treatment in present-day science that offers an evidence-based, disease-modifying treatment providing durable symptom improvement and long-term clinical tolerance, with potential against various allergic diseases.

HDM allergy is managed using measures that prevent exposure to the allergens, pharmacotherapy, and specific immunotherapy, including SLIT or SCIT [20,21]. In the present text, SLIT against HDM, for the management of allergic respiratory diseases, will be the area of our focus.

SLIT: A convenient form of AIT

SCIT for respiratory allergies was developed over a century ago. It involves weekly or biweekly administration of increasing doses by a medical professional and requires biweekly or monthly injections for maintenance. Due to this time-consuming process, a novel AIT known as SLIT was developed almost 40 years ago, which requires daily aqueous drops or tablets placed under the tongue. This convenient form of treatment can be administered by the patient at home, rather than at a clinic [20,21,24].

Mechanism of action of SLIT against HDM

**Figure 1:**
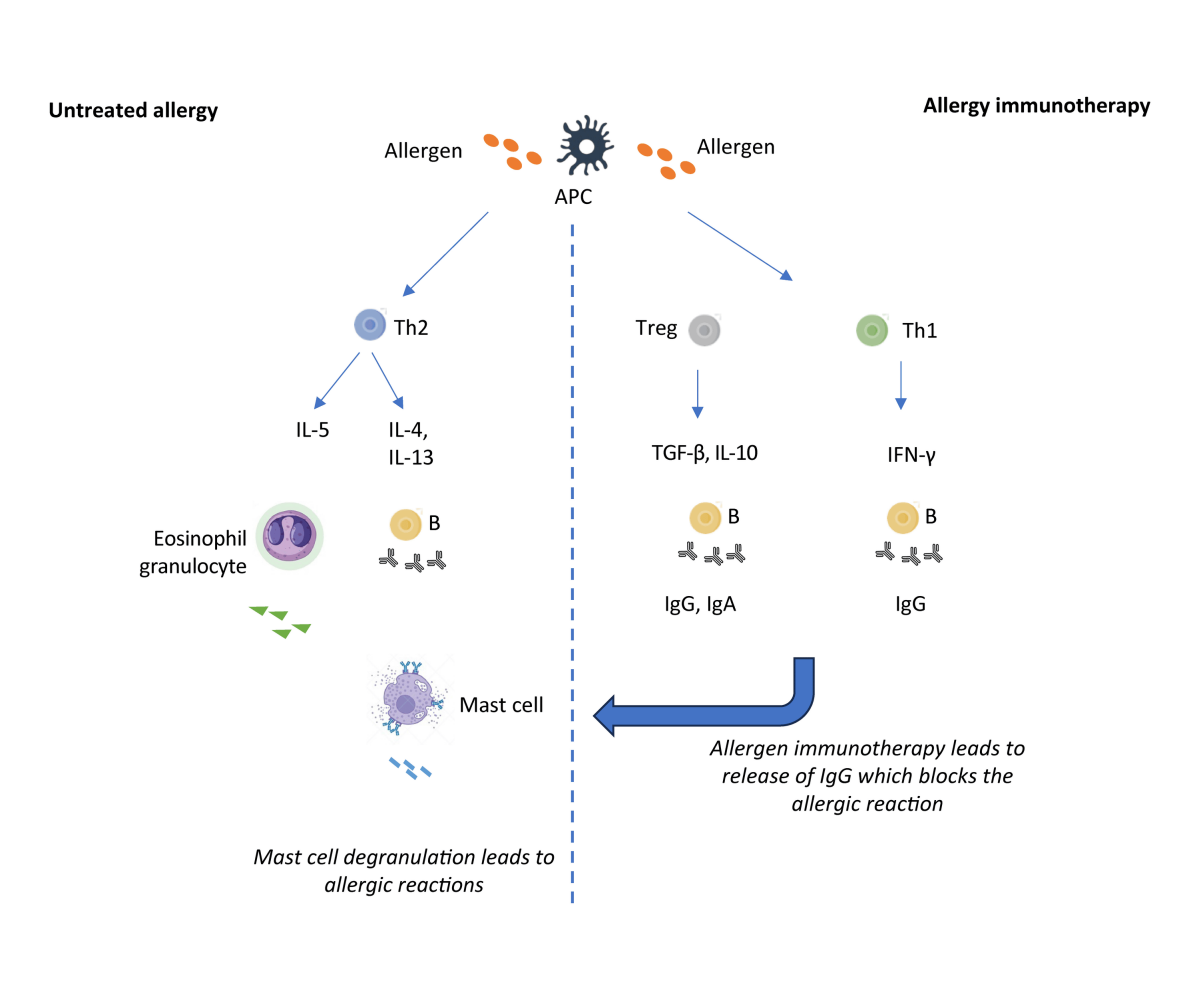
Mechanism of action of SLIT against HDM APC, Antigen-presenting cell; B, B cell; IFN-γ, Interferon-γ; IgA, Immunoglobulin A; IgG, Immunoglobulin G; IL, Interleukin; TGF-β, Transforming growth factor β; Th2, T helper 2 cell; Treg, T regulatory cell; SLIT, Sublingual Immunotherapy; HDM, House dust mite Image credit: Adapted from reference [25]

SLIT for HDM allergies is aimed at reducing allergic sensitivity by modulating the immune system’s response to HDM allergens, such as Der p 1 and Der f 1. The therapy involves placing allergen extracts under the tongue, where they interact with the immune cells in the oral mucosa. The mechanism of SLIT is outlined below and described in Figure 1.

Allergen Presentation and Initial Immune Response Occur

When administered, HDM allergens are engulfed by DCs in the oral mucosa, particularly Langerhans cells. This is followed by the migration of DCs to the regional lymph nodes and the presentation of allergens to naïve T cells, thus initiating immune modulation [5]. Induction of regulatory T cells (Tregs) follows, as naïve T cells differentiate into regulatory T cells [5] and secrete interleukin (IL)-10 and transforming growth factor β (TGF-β). These cytokines are important for suppressing allergic inflammation and redirecting the immune response toward tolerance [24]. Allergen exposure, such as to HDM, typically provokes a Th2-skewed response, characterized by the production of interleukins such as IL-4, IL-5, and IL-13 [24]. SLIT reduces the activation and cytokine production of Th2 cells, thus lowering the synthesis of allergen-specific IgE. It also increases the production of allergen-specific IgG4 and IgG1 antibodies, which act as "blocking antibodies" by competing with IgE for allergen binding. This leads to reduced allergen-mediated mast cell and basophil degranulation [20,24]. Mucosal IgA responses to SLIT administration may also contribute to the induction of tolerance [5]. This is followed by a reduction in effector cell activation. By decreasing IgE levels and Th2-mediated cytokine signaling, SLIT reduces the activation of mast cells, basophils, and eosinophils. It lowers the release of histamine and other inflammatory mediators, which are known to produce symptoms of AR and AA. Continuous SLIT leads to long-term immune tolerance. The presence of Tregs remains sustained, IgG4 levels are increased, and the shift away from the Th2 response is also durable [20,25].

Allergen Cross-Reactivity

When an antibody against one allergen binds to a similar allergen from another source, allergen cross-reactivity occurs. HDM allergens may cross-react with allergens from invertebrates, including insects, mollusks, and other mites [17,26]. Studies have reported that individuals sensitized to *D. farinae* and *D. pteronyssinus* show sensitization to HDM strains that are not present locally, indicating that cross-reactivity could play a role. Stronger cross-reactivity is noted between HDM and *A. siro* (one similar antigen) and *T. putrescentiae* (two similar antigens), compared to *L. destructor*. Cross-reactivity of around 43% has been observed between certain antigens of *D. pteronyssinus* and *B. tropicalis*. In addition, HDM and cockroach allergens cross-react in inhibition tests in a majority of sera, while cross-reactions between HDM and silverfish or chironomids have also been reported. However, in most cases of cross-reactivity, HDM remains the primary source of sensitizing allergens [26].

Efficacy of SLIT

A non-interventional, multicenter observational study included adults (18-65 years) with HDM allergy (AR alone or with AA). Patients were prescribed 30 HDM-SLIT tablets and were followed up at 1 month and 12 months after the first administration. Symptoms were categorized according to severity as “no symptoms,” “mild symptoms,” “moderate symptoms,” and “severe symptoms” for each of the nasal, eye, bronchial, and other symptoms. Among the 166 patients who completed the study, a reduction in allergy symptoms by at least one step (i.e., a decrease in at least one level of severity) was reported by 75% of patients (nasal symptoms), 62% (eye symptoms), 16% (skin symptoms), and 13% (other symptoms). Among patients with AA, a decrease of at least one step in bronchial symptoms was reported by 75% of patients. Furthermore, a 20% and 23% reduction in inhaled corticosteroids (ICS) and short-acting beta-2 agonists, respectively, was noted from baseline to the final follow-up [27].

A one-year, phase III trial compared two doses of HDM-SLIT (6 SQ-HDM and 12 SQ-HDM) with placebo among adults aged 18 to 65 years with moderate-to-severe HDM-induced AR. Both treatment groups displayed an absolute reduction in the total combined rhinitis score (TCRS) compared with the placebo group (6 SQ-HDM: 1.18, p = 0.002; 12 SQ-HDM: 1.22, p = 0.001). The treatment effect was evident from week 14 onwards. Additionally, statistically significant reductions in rhinitis symptoms and medication scores, as well as a reduced combined rhinoconjunctivitis score, were reported for both active groups compared with placebo [28].

A meta-analysis of eight clinical trials included 3,601 patients receiving 6 SQ-HDM or equivalent, 12 SQ-HDM or equivalent, 300 Index of Reactivity (IR) sublingual tablet formulation, and 500 IR sublingual tablet formulation for approximately one year. The combined symptom and medication score (CSMS) was significantly lower in all HDM-SLIT tablet groups compared with placebo (SMD: 0.28, 95% CI: 0.32 to 0.23; p < 0.01), with no significant difference between groups. Furthermore, the rhinitis symptom score (RSS) was significantly reduced in the HDM-SLIT tablet group compared to the placebo group (pooled SMD: 0.27, 95% CI: -0.32 to -0.23; p < 0.01). Significantly more patients in the HDM-SLIT group reported improvement compared with the placebo group (77.3% vs. 62.7%, p < 0.01). These findings indicate that HDM-SLIT effectively reduces rhinitis symptoms in AR patients [29].

An analysis of systematic reviews and meta-analyses reported that SLIT was effective, regardless of allergen type or age group. Furthermore, SLIT was effective in treating AR patients with HDM allergy, including when analyzed individually for adult patients and pediatric patients (effect size for symptom scores: -0.43 and -0.52, respectively) [30].

Patient Adherence and Compliance

AIT is the only disease-modifying therapy for chronic allergic conditions, and the key to achieving effectiveness is adherence to therapy in the long term (three years). Studies have reported that a treatment period of three to four years leads to continued efficacy for up to three years after discontinuation. Previous studies have reported varying rates of adherence to SLIT [31,32].

**Figure 2:**
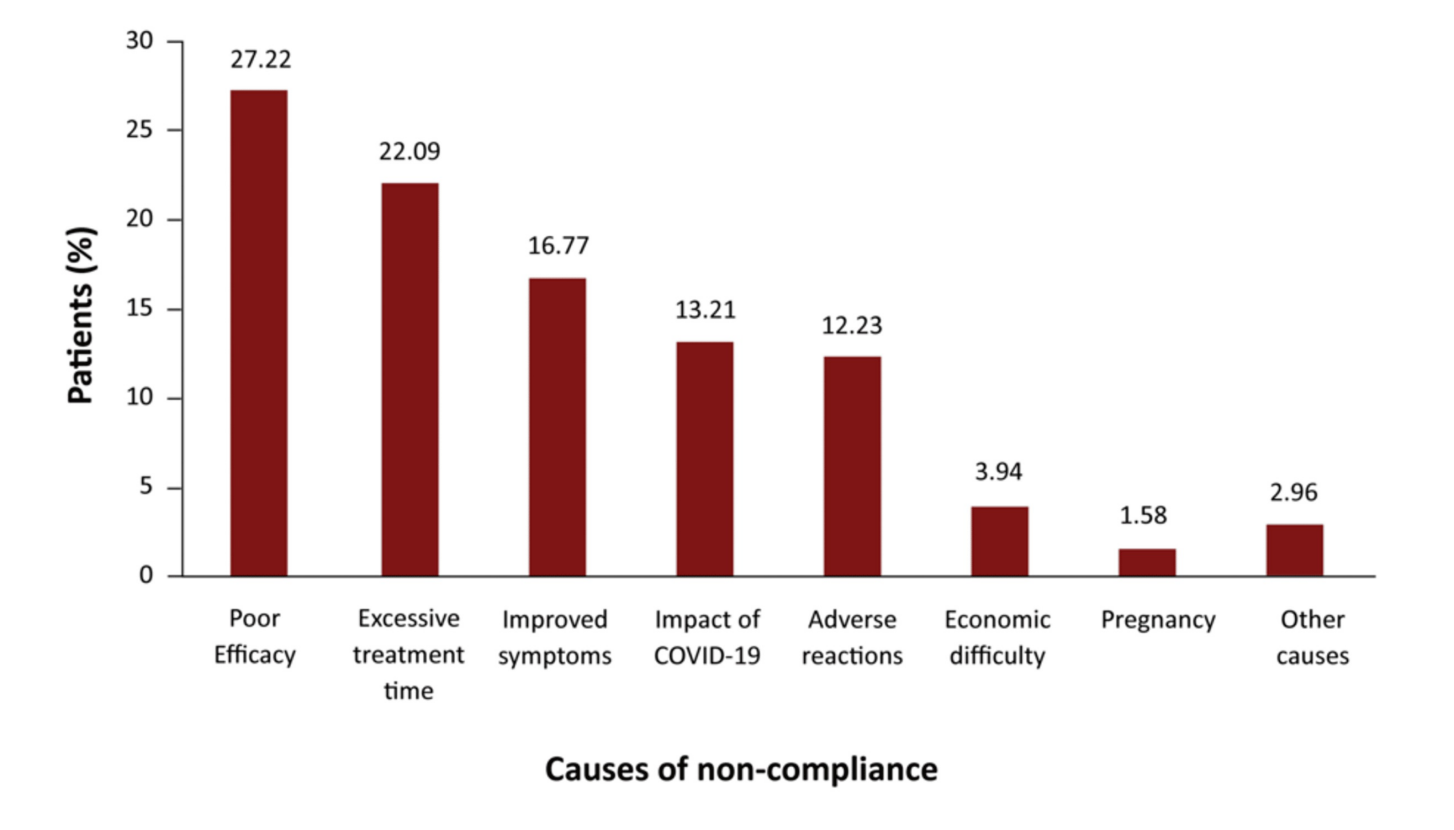
Causes of non-compliance to SCIT COVID-19, Coronavirus disease 2019; SCIT, Subcutaneous immunotherapy Image credit: Graph created using data from reference [31]

Among 507 patients aged 5-67 years, non-compliance was reported in 16.7% of patients (12.59% for children and 17.94% for adults/adolescents, p < 0.001). The causes of non-compliance are described in Figure 2 [31]. Garrido-Fernández et al. also reported high rates of adherence using the Haynes-Sackett test (91.3%) or self-reported compliance with recommended doses (86.6%). The causes of non-adherence included forgetting some doses (61.9%), lack of clinical improvement (6.2%), duration of treatment (5.2%), and clinical improvement (4.1%), among others [32]. A study among children reported a discontinuation rate of 25.7%, of whom 51.4% discontinued SLIT due to poor efficacy [33].

Despite the ease of administration of SLIT, a lower rate of adherence has been reported compared to SCIT (29.6%-33.3% at two years vs. 61.8% at two years; 9.6%-13.4% at three years vs. 37.5% at three years) for grasses. This study demonstrated that children had higher adherence rates compared to adolescents and adults [34]. In contrast, Sieber et al. demonstrated a lower dropout rate for SLIT compared with SCIT (49% vs. 66% in the SCIT natural extract group and 62% in the SCIT allergoid group) [35]. A study in Germany reported a higher dropout rate for SLIT (39% vs. 32%), but this difference was not statistically significant [36]. The findings of clinical trials are summarized in Table 1.

**Table 1:** Summary of key clinical studies evaluating efficacy of HDM-SLIT AE, Adverse effects; AR, Allergic rhinitis; HDM, House dust mite; HDM-SLIT, House dust mite sublingual immunotherapy; TEAE, Treatment-emergent adverse effect; TRCS, Total combined rhinitis score

Study design	Subjects	Intervention	Outcomes
Non-interventional multicenter, observational study [27]	183 subjects with allergic rhinitis alone or with concomitant allergic asthma	HDM-SLIT tablets for 1 year	Efficacy: Reduction in allergy symptoms by at least one step: 75% of patients (nasal symptoms), 62% of patients (eye symptoms); Reduction in bronchial symptoms by at least one step: 75% of patients. Safety: Patients with AEs: 80% (mild: 75%, moderate: 21%, severe: 2%); Anaphylactic reactions: None; Adrenaline administration: None
Randomized, parallel-group, double-blind, placebo-controlled, multinational, multisite trial [28]	Adults 18 to 65 years of age with moderate-to-severe HDM-induced AR (with or without asthma and conjunctivitis)	6 SQ-HDM, 12 SQ-HDM, or placebo for 1 year	Efficacy: Significant reductions in TRCS, combined rhinoconjunctivitis score, and rhinitis symptoms and medication scores were noted for 12 SQ-HDM. Safety: Oral pruritus: 20%; throat irritation: 14%; mouth edema: 8%; Adrenaline administration after the 12 SQ-HDM first tablet: n=1 (patient completed the trial without other AEs)
Real‐life, non‐interventional, multicenter, non‐comparative, longitudinal, prospective, descriptive study [37]	Adults 18 to 65 years of age with moderate-to-severe HDM-induced AR (with or without asthma and conjunctivitis)	6 SQ-HDM, 12 SQ-HDM, or placebo for 1 year	Safety: Adverse events possibly related to treatment: 29.4% Anaphylactic reactions: None; Adrenaline administration: None
Phase 3, open-label, single-arm, safety trial [38]	Adolescents aged 12-17 years with allergic rhinoconjunctivitis with or without asthma	12 SQ-HDM daily for 28 days	Safety: No severe local reactions; Similar safety profile for adolescents with and without asthma; no asthma-related TEAEs; Anaphylactic reactions: None; Adrenaline administration: None
Prospective, observational study [39]	521 adults and adolescents with HDM allergy who initiated HDM-SLIT	12 SQ-HDM SLIT	Safety: Serious allergic reactions: 6.8 per 1,000 person-years; Eosinophilic esophagitis: 2.3 per 1,000 person-years; Anaphylactic reactions: None; Adrenaline administration: n = 2 (frequency: 0.38%)

Patient-Reported Outcomes

**Figure 3:**
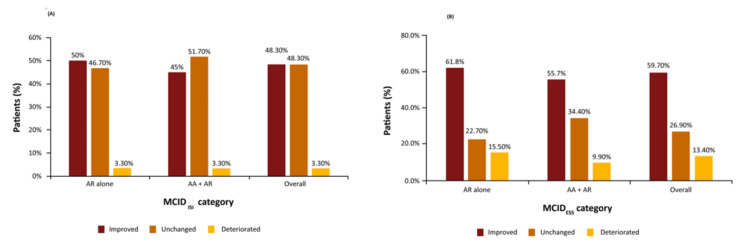
Improvement in (A) ISI score and (B) ESS score with treatment using HDM-SLIT Image credit: Graph created using data from reference [42]. For the ISI score, MCID categories were defined as follows: improved between V1 and V4 (≤ -6), unchanged between V1 and V4 (> -6 to < 6), and deteriorated between V1 and V4 (≥ 6). For the ESS score, minimal clinically important difference (MCID) categories were defined as follows: improved between V1 and V4 (≤ -2), unchanged between V1 and V4 (> -2 to < 2), and deteriorated between V1 and V4 (≥ 2). HDM-SLIT, House dust mite-sublingual immunotherapy; MCID, Minimal clinically important difference; ISI, Insomnia severity index; ESS, Epworth sleepiness scale; AR, Allergic rhinitis; AA, Allergic asthma

QoL improvements: A one-year trial of 1, 3, or 6 SQ-HDM doses reported significant improvements in the Asthma Quality of Life Questionnaire (AQLQ) compared with placebo. The 6 SQ-HDM subgroups showed greater improvement in AQLQ scores, primarily in the symptoms and activity limitation domains (differences from placebo of 0.61 and 0.52, respectively) [40]. An analysis of 11 trials of various SLIT formulations (including 2,221 patients receiving HDM-SLIT) revealed consistent and statistically significant improvements in overall Rhinoconjunctivitis Quality of Life Questionnaire (RQLQ) scores for pooled SLIT formulations versus placebo. The improvement for HDM-SLIT compared with placebo was -0.28 (p < 0.001) [41]. Jaffuel et al. evaluated the efficacy of one year of HDM-SLIT treatment among patients with persistent symptoms of moderate-to-severe HDM-induced AR, despite the use of symptom-relieving medication, or those with HDM-induced asthma-associated sleep disorders that were not well-controlled with ICS and were associated with mild-to-severe HDM-induced AR. The study reported improved scores in the Insomnia Severity Index (ISI) and the Epworth Sleepiness Scale (ESS). Overall, 48.3% and 59.7% of patients reported improvement in ISI and ESS scores, respectively (Figure 3) [42].

Cost-effectiveness analysis of SLIT: The cost-effectiveness of SLIT has been evaluated in several studies. A study conducted in Germany reported that 12-SQ HDM-SLIT reduces healthcare resource use compared to patients who do not receive any form of AIT. Specifically, a smaller proportion of patients receiving SLIT underwent hospitalization, and they had shorter lengths of stay and lower hospitalization costs compared to patients receiving SCIT. This could be attributed to a lower incidence of pneumonia and asthma exacerbations. Although the initial cost of AIT is higher, it is offset in the long term by reduced healthcare resource use [43].

Comparison With SCIT

Table 2 describes the comparison of SLIT and SCIT. The convenience of the SQ HDM-SLIT tablet is further evident, as the treatment may be initiated year-round [44,45].

**Table 2:** Comparison of SCIT and SLIT SCIT, Subcutaneous immunotherapy; SLIT, Sublingual immunotherapy

Parameter	SLIT	SCIT
Administration	Convenient form of treatment; first administration under physicians’ supervision; subsequently self-administered	Invasive form of treatment (biweekly injections at clinic/hospital followed by monthly/bimonthly injections)
Moderate-to-severe systemic adverse events	<1 reaction per 500 patients during a 3-year treatment period	1 reaction per 2,000 individual injections
Anaphylaxis risk	1 per 100 million administrations of SLIT	1 per 33,300 injections of SCIT
Local adverse effects	Frequent, minor	Frequent, minor

SLIT is considered safer than SCIT because this route of administration does not lead to detectable levels of intact allergen in the plasma, which nearly eliminates the risk of anaphylaxis. Allergens are processed by DCs, and those molecules that are not taken up by the cells undergo enzymatic hydrolysis during passage through the gastrointestinal tract. Intact allergen does not enter the vascular system to any relevant extent [24,44]. Common side effects and adverse reactions include oral pruritus (20%), throat irritation (14%), and mouth edema [37].

Practical considerations for SLIT

Place of AIT in Guidelines for AR and AA

Guidelines from the American Academy of Asthma, Allergy, and Immunology suggest that AIT (SCIT or SLIT tablets) be offered through shared decision-making to patients with moderate/severe AR who are not controlled with allergen avoidance and/or pharmacotherapy, or to those who choose immunotherapy as the preferred method of treatment (such as desiring to avoid the adverse effects, costs, or long-term use of pharmacotherapy), and/or to those who desire the potential benefit of immunotherapy to prevent or reduce the severity of comorbid conditions, such as asthma. The guidelines also suggest that SCIT or SLIT tablets should be considered for patients with controlled mild/moderate asthma with coexisting AR [46].

The Global Initiative for Asthma (GINA) guidelines recommend considering the addition of SLIT at Steps 2-4 of the treatment algorithms for adults with rhinitis who are allergic to dust mites, and who have a forced expiratory volume in one second (FEV1) of >70% [47].

European Forum for Research and Education in Allergy and Airway Diseases (EUFOREA) guidelines for AR recommend AIT for patients with uncontrolled moderate-to-severe symptoms of AR, with or without conjunctivitis, on exposure to clinically relevant allergens, and confirmation of IgE sensitization to clinically relevant allergens (via SPT or serum-specific IgE), and inadequate control of symptoms despite reliever medication and allergen avoidance measures, and/or unacceptable adverse effects of medication [42].

EUFOREA guidelines for asthma recommend AIT (SLIT) for patients with allergy-driven respiratory disease (AA with or without AR) not fully controlled by conventional treatment; those who have cognitive disturbances related to the allergy; those with AR with developing AA; and those with pollen and HDM allergies [48].

Dosing and Treatment Schedule

The dose of 12 SQ HDM-SLIT tablets is once daily. Treatment is indicated for three years to achieve a disease-modifying effect [49]. HDM SLIT tablets can be initiated at any time of the year, unlike pollen-SLIT tablets, which are initiated before the start of the pollen season. Due to the risk of potential serious adverse events, the first dose of SLIT should be given under the supervision of a trained physician in a clinical setting and must include a 30-minute observation period. Additionally, patients should be advised to recognize the symptoms of a systemic allergic reaction. In the event of such a reaction, immediate medical attention is necessary [21].

Regulatory Status of SLIT in India

Given the burden of allergic disease in India and the unmet need for HDM sensitization, SLIT presents a useful treatment option for patients with chronic allergic conditions [50]. SLIT is approved in over 40 countries, including the USA, Denmark, and Japan [51]. Interbatch variability of allergens is a known problem, and, in addition, the potency of Indian-manufactured allergen extracts of *D. pteronyssinus* and *D. farinae* is lower than that of US FDA-approved allergen extracts. In February 2024, the Central Drugs Standard Control Organization (CDSCO), India, approved the import and marketing of 12 SQ-HDM Sublingual lyophilisate (SENSIMUNE) with a clinical trial waiver, subject to a phase IV clinical trial being conducted by the pharmaceutical company [50,51]. In November 2024, it was approved for use in India for adult patients (18-65 years) diagnosed by clinical history and a positive test of HDM sensitization (SPT and/or specific IgE) with at least one of the following conditions: persistent moderate-to-severe HDM AR despite use of symptom-relieving medication, or HDM AA not well controlled by ICS and associated with mild to severe HDM AR. It is also indicated in adolescents (12-17 years) diagnosed by clinical history and a positive test of HDM sensitization (SPT and/or specific IgE) with persistent moderate-to-severe HDM AR despite use of symptom-relieving medication [52].

## Conclusions

Pharmacotherapy options for the management of allergic conditions largely provide good symptomatic relief, but the potential for adverse effects, especially with long-term use, is a barrier to achieving sustained relief. Pharmacotherapy also cannot change the natural history of these chronic allergic conditions. While AIT in the form of SCIT is effective, the practical difficulties presented above are a major limitation. SLIT offers a convenient and effective disease-modifying therapy for managing HDM-induced AA and AR, addressing the limitations of pharmacotherapy and SCIT. In view of this, SLIT presents a convenient, “at-home” form of AIT, meeting the demands of a disease-modifying therapy for chronic airway diseases. With established efficacy, safety, and guideline support, it stands out as a practical, at-home solution to improve patient outcomes and QoL. It is thus viable to explore the convenient SLIT technology for the management of bothersome HDM allergy in the right clinical setting.
